# Mortality by diseases and medical conditions in the offspring of parents with severe mental illness

**DOI:** 10.1007/s00127-019-01781-z

**Published:** 2019-10-22

**Authors:** Maria Protsenko, M. Kerkelä, J. Miettunen, J. Auvinen, M.-R. Järvelin, M. Gissler, J. Veijola

**Affiliations:** 1 Department of Psychiatry, Research Unit of Clinical Neuroscience, University of Oulu, Oulu, Finland; 2 Center for Life Course Health Research, University of Oulu, Oulu, Finland; 3 Medical Research Center Oulu, Oulu University Hospital and University of Oulu, Oulu, Finland; 4 Finnish Institute for Health and Welfare, Information Services Department, Helsinki, Finland; 5 Research Centre for Child Psychiatry, University of Turku, Turku, Finland; 6 Department of Neurobiology, Care Sciences and Society, Karolinska Institute, Stockholm, Sweden; 7 Department of Psychiatry, University Hospital of Oulu, Oulu, Finland

**Keywords:** Mortality, Natural causes, Offspring, Severe mental illness, Schizophrenia, Bipolar disorder, Depression

## Abstract

**Purpose:**

The lifespan of people with severe mental illness (SMI) is shorter compared to the general population. There might be common familial pathway leading to a high co-occurrence of somatic disorders and SMI. To study this we explored the long-term mortality for natural causes in the offspring of people with SMI.

**Methods:**

Participants were members of the Northern Finland Birth Cohort 1966 (NFBC1966; *N *= 11,325). The data on cause of deaths of the members were obtained from the Population Register Center until year 2015. The data on hospital-treated psychiatric disorders of parents were obtained from nationwide Care Register for Health Care. Cumulative incidences by age were calculated in the NFBC1966 members having a parent with SMI and those who did not have. We were able to take into account multiple confounders.

**Results:**

Of the total sample of 11,325 offspring, 853 (7.4%) died during the follow-up period, 74 (8.7%) from the study cohort and 779 (91.3%) from the comparison group. These numbers included 160 stillborn children. There were 557 cases of deaths from diseases and medical conditions and 296 deaths from external causes. The adjusted risk ratio for offspring of mothers with SMI was 1.08 (0.72–1.64), and for offspring of fathers with SMI 0.58 (0.36–0.93).

**Conclusions:**

This was the first long-term follow-up study (up to age 49) of all-cause mortality in offspring of parents with SMI. Our findings were contrary to expectations. Offspring of parents with SMI had no increased risk for dying. In fact, the risk for dying in the group of offspring of fathers with SMI was lower than in the comparison group. This study does not support the assumption of common familial pathway leading to a high co-occurrence of somatic disorders and SMI.

## Introduction

The lifespan of people with severe mental illness (SMI; schizophrenia. bipolar disorder, major depressive disorder) is shorter compared to the general population, and this is mostly due to physical illness [1]. The diseases, that are more prevalent among people with SMI compared to the general population, are nutritional and metabolic diseases, cardiovascular diseases. Patients with major mental illnesses have an increased prevalence of overweight and obesity, hyperglycemia, dyslipidemia, hypertension and smoking. The co-occurrence of mental and physical illnesses is probably due to lifestyle factors such as physical activity, diet and substance use [2]. People with SMI might also face other than disease-related factors such as poverty and reduced access to medical care, as well as adverse metabolic side effects associated with psychotropic medications [3]. There is evidence that patients with physical illness are more likely to receive delayed diagnosis, and this is due to poorer access to and quality of received health care [4].

It is widely known that mental disorders aggregate in families [5, 6]. Offspring of parents with SMI are at higher risk of developing mental illness [7]. Studies of mortality risk among offspring of mentally ill people have been conducted since the 1930s [8]. The first published studies reported no evidence for higher mortality rates in the offspring of mentally ill parents [9], whereas most studies since the 1960s have indicated greater risk for fetal, perinatal, and infant mortality associated with parental SMI, such as maternal schizophrenia. Higher mortality risks may be linked to genetic inheritance, but also to inadequate antenatal care, restricted fetal growth, medication toxicity and obstetric complications [10]. Poor health habits and decreased access to medical care or emotional, economic and social distress may also contribute to the prevalence of somatic diseases among the offspring of parents with SMI which contribute to mortality [11].

Most recent studies have identified a higher than expected risk of fetal death/stillbirth or neonatal/infant mortality in offspring of parents with mental illness [12, 13] The best evidence of increased mortality risk has come from large register-based studies conducted in Denmark [14] and Sweden [15]. However, a study from Australia [16] found no evidence of higher offspring mortality risk. In meta-analysis conducted in 2005, Webb et al. reported that most studies lack of recent evidence for mortality risk in exposed offspring beyond the first year of life [8]. The long-term outcome through their full age of risk is less known [7]. In Danish study, the mortality risk in late adolescence and early adulthood (age 16–25 years) with non-affective psychosis was elevated. The same group found out that while the risk of death from external causes was significantly increased in all age groups, the risk of death from diseases and medical conditions was not increased. Exposure during critical or sensitive periods in life, for example childhood or adolescence can have more detrimental effect than if the exposure occurs outside these time windows [17].

The objective of this study was to investigate mortality risk in offspring of parents with SMI. We will do this by exploring the possibility that there is a common familial pathway leading to a high co-occurrence of somatic disorders and SMI [18]. We studied all deaths, but specifically deaths from diseases and medical conditions. We determined if the risk of mortality increased among offspring of individuals with SMI compared to comparison group in the Northern Finland Birth Cohort 1966 (NFBC1966). Our hypothesis was that offspring of people with severe mental disorders have increased mortality risk.

## Methods

### Study design

The Northern Finland Birth Cohort 1966 (NFBC1966) is a follow-up study of offspring with expected date of birth in 1966. The data includes expected date of birth in 1966 comprising 12,068 mothers and 12,231 offspring from the provinces of Oulu and Lapland. There were 171 (1.5%) births in 1965 and 61 (0.5%) births in 1967. Our follow-up lasted from 1966 until offspring’s age of 49 years. With the help of personal identification codes given for all citizens and residents of Finland, we were able to connect register data to the NFBC1966 data set.

### Participants

**Fig. 1:**
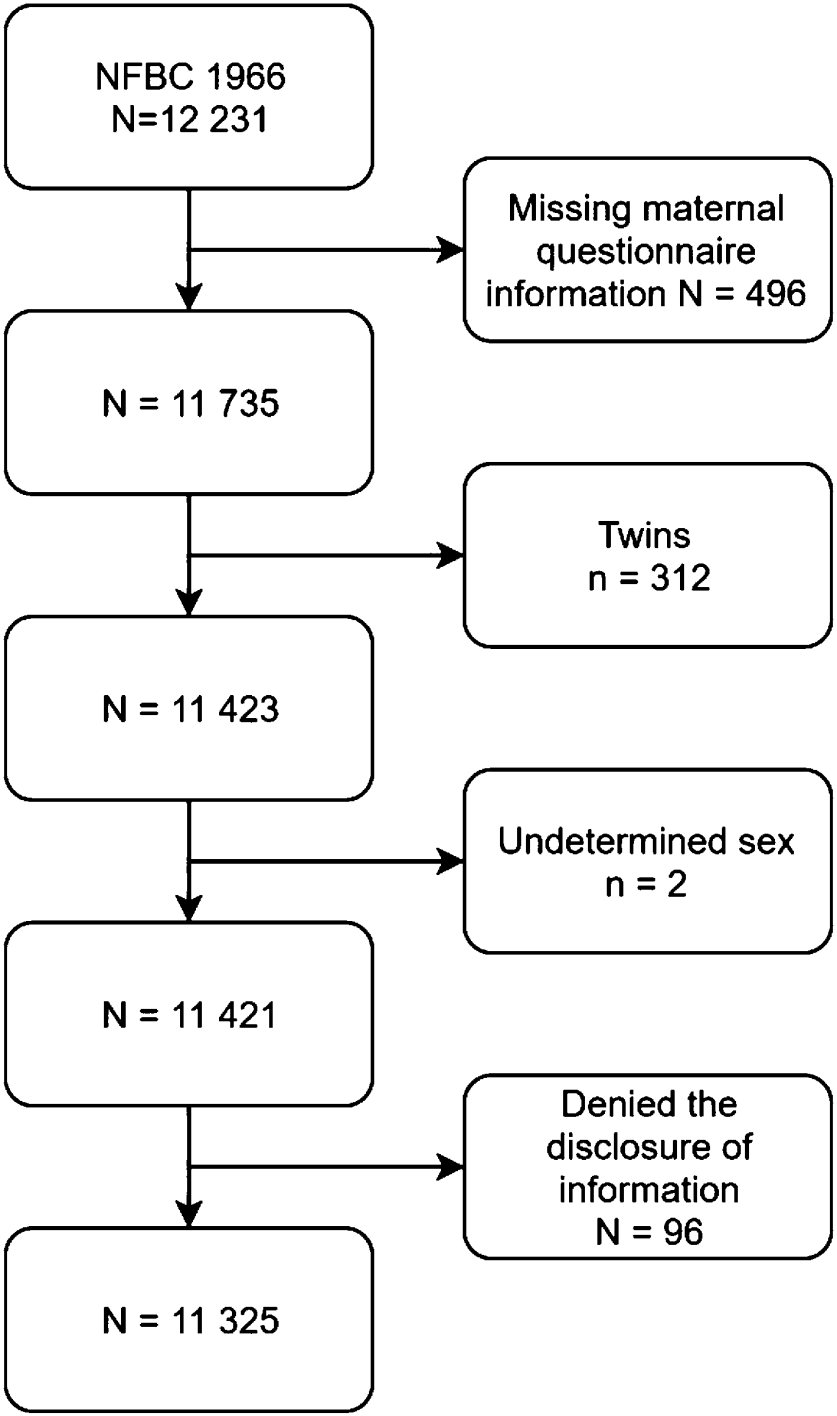
Flowchart of the study design

From our study sample we excluded 2 (0.016%) children whose sex was undetermined or could not be identified. We also excluded those 496 (4.1%) members of the NFBC1966 whose maternal questionnaire information was missing for any variable used in the present study. There were 312 (2.6%) twins which we excluded. Additional 97(0.8%) were excluded, because they denied the disclosure of information at the latest 46 year follow-up. The final study sample included 11,325 offspring (Fig. 1). This included 951 (8.4%) individuals who had a parent with SMI and 10,374 (91.6%) individuals in comparison group.

### Classification for causes of deaths

We obtained the causes of deaths of the members of the NFBC 1966 from the Statistics Finland. The causes of death were divided into two categories: deaths from diseases and medical conditions and deaths from external causes of injuries and poisoning (Table 1). The external causes of death include deaths from accidents, suicides, homicides, unspecific and missing causes of deaths, but not accidental poisoning by alcohol which are included in alcohol-related deaths under deaths from diseases and medical conditions. The classification is created and used by Statistics Finland [19]. The Finnish causes-of-death register has a good validity [20]. All causes of death and their ICD codes are double-checked regionally and at Statistics Finland.

**Table 1:** Causes of deaths from external causes of injuries and poisoning and from diseases and medical conditions. Three different ICD codes were used during the follow-up

	ICD 8* 1969–1986	ICD 9 1987–1995	ICD 10 1996–2015
Deaths from external causes of injuries and poisoning and individuals with no death certificate	291, 303, 5710, 577, E800–E999	291, 303, 3050, 3575, 4255, 5353, 5710-5713, 5770D-5770F, 5771C, 5771D, 7607A, 7795A, E800–E990	F10, G312, G4051, G621, G721, I426, K292, K70, K860, K852, P043, Q860, V01–Y89
Deaths from diseases and medical conditions	All other ICD codes	All other ICD codes	All other ICD codes

*The ICD-8 was used during 1969–1986 in Finland. The ICD-7 codes used before 1969 (in 1966-1968) were transformed to ICD-8 codes by Statistics Finland bridge coding table

### Parental SMI

The exposure of the study was parental SMI. The data of parental SMI was obtained from the Finnish Hospital Discharge Register (FHDR, currently called Care Register for Health Care, CRHC). Parents with SMI were defined as those who had received a diagnosis for any hospital treated psychiatric disorder during 1969–1982 (ICD-8 codes 290–315). We were able to study the effect of maternal and paternal SMI separately. In the whole cohort there were 400 (3.5%) mothers and 587 (5.2%) fathers with SMI. The Finnish Hospital Discharge Register is one of the oldest individual level hospital discharge registers and it has very good accuracy [21]. We also analyzed separately offspring exposed to parental alcohol and drug misuse. Due to the rarity of the events we did not make any further analyses for suggested exposure group.

### Confounders

Information on the confounders was collected from the mother by the local midwives in the antenatal clinics using a pre-defined questionnaire. The questionnaire was filled in from the 24th to 28th gestational week, but if this was not possible, the questionnaire was completed later during the pregnancy or after the delivery (10.1% of mothers).

Marital status of the mother was dichotomized into two groups: married and others (unmarried, divorced, widowed). Mothers’ educational level was categorized into three levels according to the length of educations: less than 9 years, 9–11 years, and 12 years or more. Place of birth was dichotomized into urban and rural communities. Smoking was dichotomized into two groups: smokers and non-smokers. Mothers who continued smoking in the second trimester were considered as smokers. Also sex of the offspring was used as confounder in some analysis.

### Statistical methods

We examined if offspring of people with severe mental have excess risk for mortality compared to the reference group. For this comparison we used Chi-square testing. We calculated risk ratios and used Mantel–Haenszel method for adjusted risk ratios. For adjusting we used marital status, mother’s education, mother’s smoking during pregnancy and residence of birth as confounding factors. We did not use the Cox proportional-hazards model as the assumption of proportional hazard ratios would have been violated in some of the models we used. For each exposed group (offspring of mothers with SMI, offspring of fathers with SMI and offspring of either parent or both with SMI) and unexposed group (comparison group) we calculated the risk ratios (RR) and Mantel–Haenszel risk ratios (RR_MH_) with 95% confidence intervals (CIs) using a significance threshold of 0.05. Survival curves we calculated with the Kaplan–Meier method. Statistical analyses were performed using SPSS software version 24 and RStudio version 1.2.1335.

## Results

### Descriptive analyses

Table 2 summarizes the characteristics of offspring and mothers in the NFBC1966 divided into two groups: offspring of parents with SMI and comparison group. Compared to comparison group, mothers of the offspring of parents with SMI were more likely to continue smoking during pregnancy and have lower education level.

**Table 2:** Characteristics of offspring of parents with SMI and a comparison group in NFBC1966

	Offspring of parents with SMI (*N *= 951)		Comparison group (*N *= 10,374)	
	*N*	%	*N*	%
Children’s characteristics				
Sex				
Male	475	49.9	5334	51.4
Female	476	50.1	5040	48.6
Maternal characteristics				
Smoking during pregnancy^b^				
No	761	80.0	8802	84.8
Yes	190	20.0	1572	15.2
Marital status				
Married	905	95.2	9949	95.9
Other	46	4.8	425	4.1
Education level^a^				
< 9 years	671	70.6	6880	66.3
9–11 years	247	26.0	2999	28.9
≥ 12 years	33	3.5	495	4.8
Residence				
Urban	272	28.6	3184	30.7
Rural	679	71.4	7190	69.3

^a^Significant difference between groups, *p *< 0.05

^b^Significant difference between groups, *p *< 0.001

**Fig. 2:**
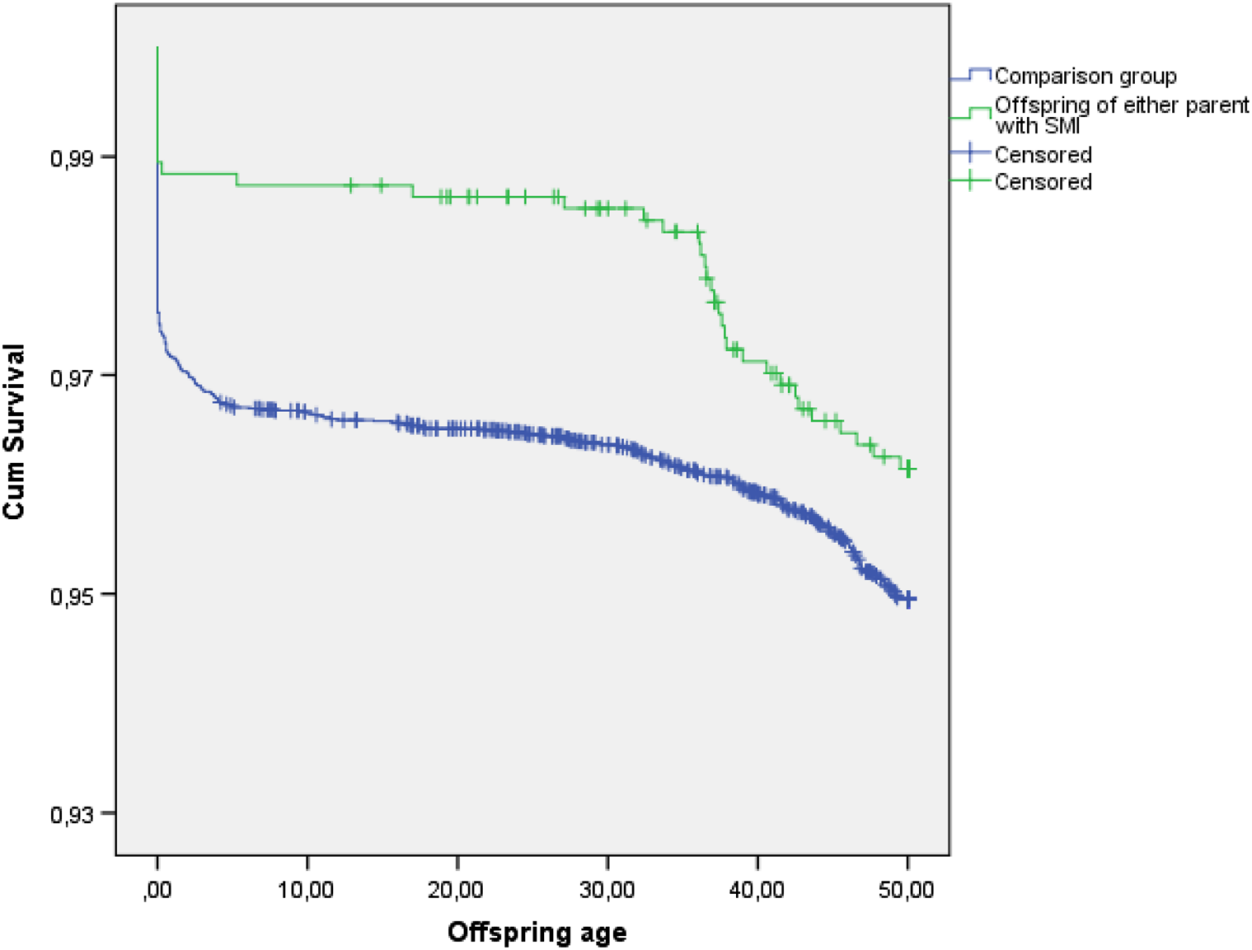
Cumulative survival of deaths from diseases and medical conditions for offspring with either parent with SMI and offspring with neither parent with SMI (comparison group) in NFBC1966

Of the total sample of 11,525 offspring, 853 (7.4%) died during the follow-up period, 74 (8.7%) from the study cohort and 779 (91.3%) from the comparison group. This number included 160 stillborn children—given the shares in the two groups. There were 557 (4.8%) deaths from diseases and medical conditions and 296 (2.6%) deaths from external causes. The results of the Kaplan–Meier survival analysis for natural causes are presented in Fig. 2. The log-rank test indicated that the survival rate was not significantly different between the groups (offspring of parents with SMI and comparison group, log rank test *p *= 0.093). The survival probability estimates at 50 years were 0.962 for offspring with at least one parent with SMI and 0.949 for comparison group.

The number of deaths from diseased and medical conditions in the NFBC1966 is presented in Table 3 as the survival rate for the 49-year follow-up period, risk ratios, and adjusted risk ratios of mortality for male and female offspring, stratified by maternal SMI, paternal SMI, and either parent with serious mental illness. The adjusted risk ratios for offspring of mothers with SMI was 1.08 (0.72–1.64), and for offspring of fathers with SMI 0.58 (0.36–0.93). The results did not change significantly using the rare events logistic regression with bias correction except for male offspring of fathers with SMI (Table 4).

**Table 3:** Deaths from diseases and medical conditions in offspring of mothers and fathers with and without SMI for all participants and separately for males and females

	Deaths from diseases and medical conditions				RR	95% CI	RR_MH_^a^	95% CI
	Yes		No					
	*N*	%	*N*	%				
All deaths								
Offspring of mothers with SMI	22	5.5	378	94.5	1.12	0.74–1.70	1.08	0.72–1.64
Offspring of fathers with SMI	17	2.9	570	97.1	0.58	0.36–0.92	0.58	0.36–0.93
Offspring of either parent with SMI	36	3.8	915	96.2	0.75	0.54–1.04	0.74	0.53–1.00
Comparison group	521	5.0	9853	95.0	1.00			
Male offspring								
Offspring of mothers with SMI	14	7.0	187	93.0	1.19	0.71–2.00	1.12	0.67–1.87
Offspring of fathers with SMI	9	3.1	281	96.9	0.53	0.28–1.02	0.52	0.27–1.00
Offspring of either parent with SMI	21	4.4	454	95.6	0.76	0.49–1.16	0.71	0.46–1.10
Comparison group	312	5.8	5022	94.2	1.00			
Female offspring								
Offspring of mothers with SMI	8	4.0	191	96.0	0.97	0.49–1.94	0.97	0.48–1.95
Offspring of fathers with SMI	8	2.7	289	97.3	0.65	0.32–1.30	0.65	0.33–1.31
Offspring of either parent with SMI	15	3.2	461	96.8	0.76	0.45–1.27	0.76	0.45–1.26
Comparison group	209	4.1	4831	95.9	1.00			

^a^Adjusted for marital status, mother’s education, mother’s smoking during pregnancy, residence of birth

**Table 4:** Deaths from diseases and medical conditions in offspring of mothers and fathers with and without SMI for all participants and separately for males and females using rare events logistic regression with bias correction

	Deaths from diseases and medical conditions							
	Yes		No					
	*N*	%	*N*	%	OR	95% CI	Adjusted OR^a^	95% CI
All deaths								
Offspring of mothers with SMI	22	5.5	378	94.5	1.15	0.74–1.79	1.10	0.71–1.70
Offspring of fathers with SMI	17	2.9	570	97.1	0.58	0.35–0.95	0.57	0.35–0.93
Offspring of either parent with SMI	36	3.8	915	96.2	0.75	0.53–1.06	0.73	0.52–1.03
Comparison group	557	4.9	10,768	95.1	1.00		1.00	
Male offspring								
Offspring of mothers with SMI	14	7.0	187	93.0	1.28	0.74–2.23	1.15	0.66–2.01
Offspring of fathers with SMI	9	3.1	281	96.9	0.54	0.28–1.06	0.53	0.27–1.04
Offspring of either parent with SMI	21	4.4	454	95.6	0.76	0.48–1.20	0.71	0.45–1.12
Comparison group	333	5.7	5476	94.3	1.00		1.00	
Female offspring								
Offspring of mothers with SMI	8	4.0	191	96.0	1.05	0.51–2.15	1.03	0.50–2.13
Offspring of fathers with SMI	8	2.7	289	97.3	0.68	0.33–1.39	0.68	0.33–1.38
Offspring of either parent with SMI	15	3.2	461	96.8	0.77	0.45–1.32	0.77	0.45–1.31
Comparison group	224	4.1	5292	95.9	1.00		1.00	

^a^Adjusted for marital status, mother’s education, mother’s smoking during pregnancy, residence of birth

## Discussion

### Main findings

This was the first long-term follow-up study (up to age 49) of mortality to medical causes in offspring of parents with SMI. Our findings did not support our hypothesis of higher mortality from diseases and medical conditions in offspring of parents with SMI. The findings did not either support the assumption of common familial pathway leading to a high co-occurrence of somatic disorders and SMI in offspring of parents with SMI.

### Comparison to earlier studies

Most studies focusing on mortality in children of parents with SMI have focused on fetal, perinatal or infant mortality, but there are two earlier studies that have included longer follow-up periods up to age 50 [22] or over 50 years [23]. The previous suggested that there is increased all-cause mortality risk from late adolescence until middle age. The latter did not find association between parental SMI and offspring all-cause mortality, however, the study was rather old. Recent studies with shorter follow-up period, mostly including infant and childhood mortality, have found positive association between parental SMI and offspring mortality [8, 10].

The effect of parental SMI to offspring disease mortality is topic that has not been studied much. An increased mortality risk by diseases and medical conditions could be due to cardiometabolic disorders. Andreassen et al. [24] have found shared loci in schizophrenia and cardiovascular risk factors. In addition, Spelman et al. [25] have suggested impaired glucose tolerance in first-degree relatives of schizophrenic patients compared to healthy controls. Shared environmental factors may also contribute to the elevated prevalence of physical diseases in the relatives of people with SMI, such as poor health habits and reduced access to or compliance with medical care. This possibility is supported by Rudisch and Nemeroff [26] showing association between cardiometabolic risk and depression. Caretaker burden is one environmental factor increasing the prevalence of cardiometabolic disorders among offspring of parents with SMI [27]. Additionally, studies have reported higher emotional, economic and social distress among relatives of people with SMI [28, 29].

#### Effects of paternal SMI

Between 1968 and 1980, four studies have included paternal exposure on results of offspring mortality risk [8, 9, 23, 30, 31]. The results of those studies were controversial. One study suggested that there was increased risk among offspring of fathers with SMI (also among offspring of mothers with SMI) [31]. Other study suggested that the sex of affected parent had no effect on survival rate either for male or female offspring of parents with SMI and that the offspring of schizophrenic parents have increased survival during the first year of life [9]. The other studies found that there was no evidence of increased mortality among offspring of parents with SMI [23, 30]. Webb et al. reported highest risk in all-cause mortality associated with paternal disorder occurred during the post neonatal period. They found one high-risk group: 5–15 years olds whose fathers were admitted for alcohol-related disorders. However, during school attendance years, risks specific to all other types of paternal disorder were not elevated [10]. Liu et al. [12] also reported elevated risk for all-cause mortality for offspring born to schizophrenic fathers. These results are contrary with our findings, however, in our results the risk was elevated only when examining the deaths from external causes. The finding for these deaths was in line with earlier research, but was not the topic of present study. The risk was lower when examining the causes of death related to diseases and medical conditions and this result is maybe because lacking paternal attendance in offspring’s life. Unfortunately we did not have any data on paternal attendance.

#### Effects of maternal SMI

When studied previous literature linking maternal SMI to offspring mortality risk, the most recent studies have documented an increased risk of death in exposed offspring. In Finland (2008), the results have indicated that exposure to maternal psychotic illness is associated with over two-fold risk of dying [22]. The meta-analysis, conducted by Webb et al., found almost two-fold relative risk for fetal death/stillbirth among offspring of affected mothers, which is comparable in magnitude to that observed for maternal smoking during pregnancy [8, 32]. In Asian society, Chen et al. [33] reported that offspring of parents with schizophrenia and affective disorders, mortality risks were higher among offspring with maternal mental illness than those with paternal mental disease. However, their follow-up was only from birth until 5 years of age. These findings are in line with our results. We found no statistically significant results linking maternal mental illness to offspring mortality. However, we found that offspring of mothers with SMI smoke more likely during pregnancy. This is in line with previous research.

When studying mortality risk in early adulthood from 16 to 25 years among offspring of mothers with psychotic disorders, Webb et al. found 1.56-fold risk of dying [10]. We found during our 49 year follow-up time 1.07-fold mortality risk for diseases and medical conditions, but the result did not reach statistical significance. There are several factors that may increase mortality risk in adult offspring of mothers with SMI compared to comparison group. One is that offspring of parents with SMI are in higher risk of developing mental illness [7] and severe mental illnesses are associated with increased mortality from natural causes [34].

#### Effects of offspring sex

Suvisaari et al. [22] reported no sex differences in mortality from natural causes between the groups, but males had a significantly higher mortality risk from external causes. None of their variables predicted mortality from diseases and medical conditions. Our study resulted higher risk ratios for female offspring, but the results were statistically insignificant. This may be due to small sample size.

### Strengths

There are several strengths in the present study. First, the study was long-term with follow-up of the offspring until 49 years. Only two earlier studies have been able to conduct such extensive follow-up period. Most studies have focused on infancy and early childhood. Second, our study focused on mortality from diseases and medical conditions in the offspring. Majority of earlier studies have studied all-cause mortality, but not separated different causes-of-death. Of the studies separating causes most have focused on external causes and not on deaths from diseases and medical conditions.

Third strength was the possibility to link two reliable nationwide registers, namely The Finnish Hospital Discharge Register (FHDR) and the Causes-of-death Register at Statistics Finland. Reporting is obligatory in Finland and the quality of these registers has been shown to be good. The fourth strength was the prospectively collected data set of the NFBC1966. The follow-up started during pregnancy, and we were able to use multiple confounders including maternal smoking during pregnancy and socio-economic factors. Many of the recent register-based studies investigating mortality risks among offspring of parents with SMI have declared as limitation the inability to adjust the potentially important confounder of socioeconomic status [13, 14, 35].

The fifth strength was our ability to analyze separately the effect of maternal and paternal SMI. We had a good validity in our data set for statistical analyses. With adjusted risk ratios we could make comparisons between exposed and unexposed groups separately when maternal and paternal SMI was studied. Most previous studies have focused on the effect of maternal SMI, but we could also add the paternal SMI to our analyses.

### Limitations

However, our study had some limitations. First, although a large birth cohort was studied, the rarity of premature deaths among exposed offspring reduced the statistical power of the study. This led to rather wide confidence intervals. In addition, some of the data were collected via questionnaire so those variables may not be as reliable compared to register data.

Second, we were not able to study parental SMI before pregnancy, during pregnancy or in the infancy of the offspring. This is due to lacking data from year 1966 to 1968 as the FHDR did not have a complete registration of personal identification codes before 1969. This means that there was a 2–3 years gap on parental diagnoses. Third, the outcome was rare so there is a possibility of false negative finding. Fourth, we had no information on offsprings’ placement during childhood. In other words, we did not know if the child was living with their mother or father or placed out of home. However, there were relatively same amount of offspring having both parents in both groups (offspring of parents with SMI and comparison group).

### Conclusions

This was the first recent long-term follow-up study (up to age 49) of mortality from diseases and medical conditions in offspring of parents with SMI. Our findings were contrary to expectations. Offspring of parents with SMI had no increased risk for dying from diseases or medical conditions. In fact, the risk for dying in the group of offspring of fathers with SMI was lower than in the comparison group. This study does not support the assumption of common familial pathway leading to a high co-occurrence of somatic disorders and SMI.
